# Principle in Practice

**Published:** 1894-08

**Authors:** 


### Editorial.

### PRINCIPLE IN PRACTICE.

It is sometimes difficult to carry ethical laws into the professional daily work, and in some of the important relations of the business and political world it seems wholly neglected. The somewhat trite expression that "business is business," meaning thereby that the altruistic idea is always to be subordinated to the selfish advancement of the individual, has, it is feared, become the motive force of large combinations of men to the destruction of the moral law supposed to be necessary for the government of the individual.

The effort has always been, in professions, to eliminate this idea and give a higher tone to the relations between the lawyer and his client, the divine and his congregation, the doctor and his patient. The honorarium of the physician is time-honored, and while it has practically no existence in this country it is still an active force in the Old World, and in some nationalities to send a bill for services rendered is regarded as wholly unprofessional.

While the activities of the world have made inroads in these usages, and modified the ethical code to bring it more in harmony with business ideas, the impress of a higher moral law for the government of professional men still remains to modify the selfish side of human ambition and human greed.

While it is true none of the callings named will work without remuneration, they each, in their special way, labor without a hope of reward to an extent that the outside world knows nothing of, nor, it may be presumed, would it appreciate it. The sentiment of helpfulness to your neighbor will never die out while professions live, however much individuals belonging to them may endeavor to turn the current of thought in other directions.

We are not so much concerned in this article with what may or can be done to help suffering humanity without hope of reward, as we are with the ethical upbuilding of the profession in which we find ourselves workers.

Dentistry began as a trade and the instincts of trade have closely adhered to it, and in the evolution towards a profession it has been found difficult, if not impossible, to remove this taint and bring it more into line with the professional spirit. That much has been gained must be acknowledged, but that more is needed must be apparent to every active observer.

The higher development can only come through a more elevated standard of education, both primary and professional, but much may be done to hasten a better conception by personal influence, one upon the other.

One of the most encouraging evidences of a change for the better is manifest in the higher tone of dental journals. Formerly, indeed less than a decade, the conductors of these periodicals were satisfied not to lead but to follow, and this course, unfortunately, led most of them into a lower standard than the profession then occupied. This may be accounted for upon the fact that dental journalism had its origin in trade and was not able to rise above its source. That the publishers of these periodicals have seen the necessity for a change of tactics, and have permitted a large margin of liberty to their editors, is a happy omen of a better state of things, but even here much remains to be desired. The character of some of these is by no means up to the standard of dignity always to be maintained in professional work, whether in the office or at the desk.

It is trite to say, but nevertheless true, that professions are made up of individual elements, and in proportion as these become purified will the entire body advance towards perfection. That we have reached this desirable stage in our progress cannot be said with truth. There is still a tendency to accomplish ends by means not altogether creditable and oftentimes injurious.

Perhaps this is more noticeable in the effort of the few to appropriate the work of others without giving due credit or no credit at all. This tendency to plagiarize was alluded to in a former article, and need only be mentioned here as one of the evils of serious magnitude infesting professional work.

The one great obstacle to progress is the want of moral fibre to withstand the temptations constantly presented in practice. These come in various forms,—the wrong done to patients by not doing the best possible for them; the shameful neglect so often witnessed in performing the daily operations; the lack of that necessary care in sterilization, and indifference to the possibility of spreading disease through uncleanliness; the effort to perform unnecessary and expensive operations for the purpose of personal credit, and, above all, in the use of medicaments, compositions of which the operator is ignorant.

This latter offence has become so serious that a determined stand is necessary, or the dental profession will become a by-word and reproach.

The word "obtundent" has become offensive in the ears of those anxious for better things. Empirical preparations are to-day endangering the health and even the lives of dental patients, but more than this, they are sapping the moral vitality of the profession.

The time has come for a decided position on this question by all the organizations of the world, as the evil is not confined to any one country. The lack of moral strength is to be pitied that leads a dentist to use these unknown compounds, under the weak plea "that my neighbor uses them and, if I am to live, I must."

That ignorance lies at the foundation of this practice must be apparent, ignorance of the possibly dangerous results when injected hypodermically, and ignorance of the pathological laws governing sensibility in tooth-structures.

We have not the space to enter into this branch of the subject, and must be content with asserting, somewhat dogmatically, that the entire obtunding of sensation means a loss of tone and possible destruction of the conductors of sensation. Illustrations of this may be found in the devitalization of pulps, and in necrosis from hypodermic injections.

The moral degradation of the professional spirit is the more serious evil. There is but one course to pursue in this, as well as other matters of an ethical character, and that is, to walk closely to the path which leads to the best interest of those with whom we have to deal, and in thus doing we will be able to keep in the road that leads directly to the higher moral law enunciated nineteen hundred years ago.

